# The HDAC inhibitor domatinostat induces type I interferon α in Merkel cell carcinoma by *HES1* repression

**DOI:** 10.1007/s00432-023-04733-y

**Published:** 2023-04-18

**Authors:** Nalini Srinivas, Lina Song, Kuan Cheok Lei, Jan Gravemeyer, Frauke Furtmann, Thilo Gambichler, Jürgen C. Becker, Ashwin Sriram

**Affiliations:** 1 Department of Translational Skin Cancer Research (TSCR), German Cancer Consortium (DKTK), Partner Site Essen, University Medicine Essen, and German Cancer Research Center (DKFZ), Heidelberg, Germany; 2 Department of Dermatology, Zhongshan Hospital, Fudan University, Shanghai, China; 3 Skin Cancer Center, Department of Dermatology, Ruhr-University Bochum, Bochum, Germany; 4 Department of Dermatology, University Hospital Essen, Essen, Germany

**Keywords:** Merkel cell carcinoma, Histone deacetylase inhibitor, Interferons, HES1, Interferon-stimulated genes

## Abstract

**Background:**

Class I selective histone deacetylase inhibitors (HDACi) have been previously demonstrated to not only increase major histocompatibility complex class I surface expression in Merkel cell carcinoma (MCC) cells by restoring the antigen processing and presentation machinery, but also exert anti-tumoral effect by inducing apoptosis. Both phenomena could be due to induction of type I interferons (IFN), as has been described for HDACi. However, the mechanism of IFN induction under HDACi is not fully understood because the expression of IFNs is regulated by both activating and inhibitory signaling pathways. Our own preliminary observations suggest that this may be caused by suppression of HES1.

**Methods:**

The effect of the class I selective HDACi domatinostat and IFNα on cell viability and the apoptosis of MCPyV-positive (WaGa, MKL-1) and -negative (UM-MCC 34) MCC cell lines, as well as, primary fibroblasts were assessed by colorimetric methods or measuring mitochondrial membrane potential and intracellular caspase-3/7, respectively. Next, the impact of domatinostat on *IFNA* and *HES1* mRNA expression was measured by RT-qPCR; intracellular IFNα production was detected by flow cytometry. To confirm that the expression of IFNα induced by HDACi was due to the suppression of HES1, it was silenced by RNA interference and then mRNA expression of *IFNA* and IFN-stimulated genes was assessed.

**Results:**

Our studies show that the previously reported reduction in viability of MCC cell lines after inhibition of HDAC by domatinostat is accompanied by an increase in IFNα expression, both of mRNA and at the protein level. We confirmed that treatment of MCC cells with external IFNα inhibited their proliferation and induced apoptosis. Re-analysis of existing single-cell RNA sequencing data indicated that induction of IFNα by domatinostat occurs through repression of HES1, a transcriptional inhibitor of *IFNA*; this was confirmed by RT-qPCR. Finally, siRNA-mediated silencing of HES1 in the MCC cell line WaGa not only increased mRNA expression of *IFNA* and IFN-stimulated genes but also decreased cell viability.

**Conclusion:**

Our results demonstrate that the direct anti-tumor effect of HDACi domatinostat on MCC cells is at least in part mediated via decreased HES1 expression allowing the induction of IFNα, which in turn causes apoptosis.

## Introduction

Merkel cell carcinoma (MCC) is an aggressive skin cancer with a neuroendocrine differentiation. Its carcinogenesis is associated either with the integration of the Merkel cell polyomavirus (MCPyV) into the host genome and the constitutive expression of viral early genes, i.e., small and large T antigen, or exposure to ultraviolet radiation mutagenesis, both resulting in an explicit immunogenicity of this cancer (Becker et al. 2017; Gravemeyer et al. 2022; Harms et al. 2022). Thus, immunotherapy with immune checkpoint inhibitors (ICI) became the first choice to treat patients with advanced disease (Gauci et al. 2022). However, a significant proportion of MCC patients does not show durable tumor regression under immunotherapy. This circumstance is explained by multiple immune escape mechanisms of MCC cells. For example, we have shown that MCC cells reduce the surface expression of major histocompatibility complex (MHC) class I antigen processing and presentation machinery (APM) proteins through epigenetic changes, which in turn is associated with defective recognition by specific anti-tumor T cells (Ritter et al. 2017). Importantly, the functional loss of MHC class I cell surface expression can be restored by both broad-spectrum and class I selective histone deacetylase inhibitors (HDACi) (Mazziotta et al. 2022; Ritter et al. 2017). We previously reported that the isotype-specific HDACi, domatinostat, which selectively inhibits HDAC 1, 2, and 3 not only increases MHC class I surface expression in MCC cells by restoring the APM, but also exerts direct anti-tumoral effect by apoptosis induction (Song et al. 2021). Both phenomena could be due to induction of type I interferons (IFNs), as has been described for HDACi (Kotredes and Gamero 2013; Coomans de Brachene et al. 2018; Peteranderl and Herold 2017). Acetylation of histones and other proteins has been implicated in regulating both expression of and cellular response to IFNs (Nusinzon and Horvath 2003; Lu et al. 2019). However, particularly the mechanisms of induction of IFN expression by HDACi are not fully understood. The fact that the expression of IFNs is regulated both by activating and inhibitory signaling pathways does not make the mechanism any easier to uncover (Ivashkiv and Donlin 2014; Chen et al. 2017). For example, toll-like receptor (TLR) signaling and IFN regulatory factors (IRF) 3 lead to the expression (Honda and Taniguchi 2006), whereas the basic helix–loop–helix transcription factor hairy and enhancer of split 1 (HES1) functions as a homeostatic negative regulator of IFNs (Ning et al. 2019). Indeed, HES1 suppresses both the production of type I IFNs and the expression of IFN-stimulated genes. HES1 does not suppress IFN expression directly, but rather by inhibiting the toll-like receptor signaling adaptor molecule WD-repeat and FYVE domain containing protein 1 (WDFY1) (Ning et al. 2019). Here, we report that domatinostat induces expression of IFNα, in MCC cells by suppression of HES1, thereby promoting innate immunity, ultimately resulting in the death of the cancer cell.

## Materials and methods

### MCC cell lines and cell culture

MCPyV-positive, WaGa and MKL-1, and -negative, UM-MCC 34, MCC cell lines have been previously described (Schrama et al. 2019; Fan et al. 2018). All the cell lines were cultured under standard conditions (37 °C, 5% CO_2_) in RPMI 1640 medium (PAN-Biotech, Aidenbach, Germany) supplemented with 10% fetal bovine serum (FBS; PAN-Biotech) and 1% penicillin–streptomycin (P/S; PAN-Biotech). Primary skin fibroblasts isolated from healthy skin (F1.15) were cultured in DMEM medium (PAN-Biotech) and DMEM/F12 (1:1) medium (Lonza, Cologne, Germany), supplemented with 10% FBS and 1% P/S under standard conditions (Fan et al. 2021). The cell lines were regularly tested to ensure the absence of mycoplasma and their identity was verified by DNA finger printing.

### Domatinostat and interferon treatment

The cell lines were treated with Domatinostat (4SC AG, Planegg-Martinsried, Germany) as described earlier (Song et al. 2021) and human IFNα 2a (Gibco, Thermofischer Scientific, Germany), independently. Briefly, the cells were seeded in 6-well plates at a concentration of 1 × 10^6^ cells/ml. A stock concentration of 50 mM domatinostat was prepared by dissolving in DMSO (PanReac AppliChem, Darmstadt, Germany) and a working concentration of 10 mM domatinostat, dissolved in the respective culture medium (without FBS and P/S), was used for further experiments. The cells were treated at a final concentration of 2.5 µM and 5 µM Domatinostat each per well for 24 h at 37 °C. After, IFNα 2a was added to achieve the following concentrations: 781.25, 3,125, 12,500, and 50,000 ng/ml, and the cells were incubated for 7 days at 37 °C.

### Cell viability assays

CellTiter 96 Aqueous One Solution Cell Viability Assay (Promega, Mannheim, Germany) was used to measure the viability of MCC cells and primary fibroblasts. The cells were cultured in 96-well assay plates 24 h prior to treatment. Cells were treated with domatinostat and IFNα 2a at the indicated concentrations in triplicates, respectively. After treatment, 20 µl of CellTiter 96 Aqueous One Solution Cell Proliferation Assay reagent was added to each well. The cells were then incubated for 120 min at 37 °C before absorbance was measured at 490 nm using a Spectramax microplate reader (Molecular Devices, San Jose, CA). In addition, the number of viable cells was also manually quantified based on Trypan blue exclusion assay.

### Apoptosis assays

To determine cellular apoptosis, the NucView 488/MitoView 633 Apoptosis assay kit (Biotium, Fremont, CA) was used according to the manufacturer’s instructions. Viable and apoptotic cells were visualized by fluorescence microscopy (Zeiss Axio Observer Z1, Oberkochen, Germany) and quantified by flow cytometry. Data were analyzed using FlowJo version 10 software (TreeStar, Sunnyvale, CA, USA).

### Intracellular IFNα staining

For intracellular IFNα staining, both control and domatinostat-treated cells were incubated with Brefeldin A (GolgiPlug, 1:1000, BD Biosciences, San Jose, CA, USA) for 6 h at 37 °C. The cells were fixed using 4% formalin for 20 min at room temperature, permeabilized using 0.05% Triton-X in PBS for 20 min at 4 °C and stained with anti-IFNα antibody (IFNα Antibody, anti-human, PE, Miltenyi Biotec, Germany) for 24 h in the dark at 4 °C. The cells were analyzed by flow cytometry (Cytoflex, Beckman Coulter, US) and the fluorescence intensity was measured using CytExpert software (version 2.1, Beckman Coulter). Cell debris was excluded from the analysis based on scatter signals.

### siRNA-mediated knockdown of HES1

For HES1 knockdown, liposome-based reverse transfection was performed using predesigned dicer-substrate small interfering RNA (DsiRNA; TriFECTa kit, Integrated DNA Technologies, Leuven, Belgium) and Lipofectamine™ RNAiMAX (Thermofischer, Dreieich, Germany) following manufacturer’s instructions. The TriFECTa kit consists of 3 DsiRNAs that are 27mer duplex RNAs, targeting different regions of the gene, along with a universal negative control duplex, which targets a site that is absent from human genomes. The siRNA sequences are shown in Table 1. In reverse transfection, the complexes are prepared inside the wells, after which the cells and medium are added. In each well of a 6-well plate, DsiRNA (10 nM) was diluted in 500µL Opti-MEM reduced serum medium (Gibco, Fischer Scientific, Schwetrte, Germany), gently mixed and incubated at room temperature for 5 min. 5 µl Lipofectamine™ RNAiMAX was added to each well containing the diluted DsiRNA molecules, gently mixed and incubated for 10 min at room temperature. WaGa cells at a concentration of 0.5 × 10^6^ cells/ml were added to the DsiRNA–lipofectamine complex, and the experiment was carried out in triplicates. A final volume of 3 ml per well was achieved by adding RPMI1640 medium (PAN-Biotech) without any serum or antibiotics. The cells were incubated for 6 h at 37 °C, after which 10% FBS was added and further incubated for 12, 24, 36, 48, 60, and up to 72 h. Cell viability and apoptosis assays were also performed for siRNA-mediated HES1 knockdown cells.

**Table 1:** HES1 dicer-substrate small interfering RNA (DsiRNA) sequences

DsiRNA	Sequence
DsiRNA–hs.Ri.HES1.13.1	5′- ACCGGAUAAACCAAAGACAGCAUCT-3′
	3′- UGUGGCCUAUUUGGUUUCUGUCGUAGA -5′
DsiRNA–hs.Ri.HES1.13.2	5′- CGGACUCUAAACAGGAACUUGAATA- 3′
	3′- UUUUCUGCUUCUCGUUCUUAUUUACUU-5′
DsiRNA–hs.Ri.HES1.13.3	5′- CGGACUCUAAACAGGAACUUGAATA- 3′
	3′- AGGCCUGAGAUUUGUCCUUGAACUUAU- 5′

### Quantitative real-time reverse transcriptase PCR

RNA was isolated using PeqGOLD Total RNA kit (VWR/Peqlab, Erlangen, Germany) and transcribed into cDNA using the Super Script IV Reverse Transcriptase (Thermo Fischer Scientific, Dreieich, Germany) according to the manufacturer’s instructions. Quantitative real-time reverse transcriptase PCR (RT-qPCR) was performed on the CFX Real-Time PCR system (BioRad Laboratories, Düsseldorf, Germany). *RPLP0* served as endogenous control, *HES1, IFNA, IFNB, IFNAR1, IFNAR2, JAK2,* and *IRF3* mRNA expression was detected using SYBR green assays, and relative quantification was calculated by the 2^−ΔΔCq^ method using the respective untreated control or as indicated. Primer sequences are given in Table 2.

**Table 2:** List of gene primers

Gene	Forward primer (5′ → 3′)	Reverse primer (5′ → 3′)
*HES1*	TCAACACGACACCGGACAAAC	ATGCCGGGAGCTATCTTTCTT
*IFNA*	GCCTCGCCCTTTGCTTTACT	CTGTGGGTCTCAGGGAGATCA
*IFNB*	ATGACCAACAAGTGTCTCCTCC	GGAATCCAAGCAAGTTGTAGCTC
*IFNAR1*	AACAGGAGCGATGAGTCTGTC	TGCGAAATGGTGTAAATGAGTCA
*IFNAR2*	TCATGGTGTATATCAGCCTCGT	AGTTGGTACAATGGAGTGGTTTT
*JAK2*	TCTGGGGAGTATGTTGCAGAA	AGACATGGTTGGGTGGATACC
*IRF3*	AGAGGCTCGTGATGGTCAAG	AGGTCCACAGTATTCTCCAGG
*RPLP0*	CCATCAGCACCACAGCCTTA	GGCGACCTGGAAGTCCAACT

### Single-cell RNA sequencing analysis

The results of single-cell sequencing analysis had been reported before, but the data had been re-analyzed here (Song et al. 2021). Briefly, WaGa cells treated with either domatinostat (2.5 µM, 24 hours at 37°C) or solvent control were subjected to single-cell RNA sequencing (Song et al. 2021). The Cell Ranger Single Cell Software Suite, version 2.1.1 (http://10xgenomics.com/) was used with default settings to separately align cDNA reads to the hg19 human genome. Data were then aggregated into one file using the Cell Ranger, loaded into R and processed using Seurat framework. In order to remove low quality, cells were only kept for downstream analysis if they expressed between 1000 and 5000 genes, 1000 and 3000 UMIs, more than 65 housekeeper genes and if the expression of mitochondrial genes comprised less than 10% of all UMIs in a cell. Afterward, normalization and computation of dimension reductions was performed inside Seurat. The data was re-analyzed, accounting for the cell cycle effects during normalization, and the expression of *HES1* was investigated within the data.

### Statistical analysis

Statistical analyses were performed using GraphPad Prism 8.0 software (GraphPad software, San Diego, A). Experiments containing two groups were analyzed using the Mann–Whitney U test. Experiments containing more than two groups were analyzed using the Kruskal–Wallis test, an unpaired nonparametric ANOVA. A *p*-value of less than 0.05 was considered significant; the respective *p*-values were indicated in the figure as follows: *, *p* < 0.05; **, *p* < 0.01; ***, *p* <0.001; ****, *p* < 0.0001.

## Results

### Induction of IFNA expression in MCC cells by treatment with domatinostat

**Fig. 1:**
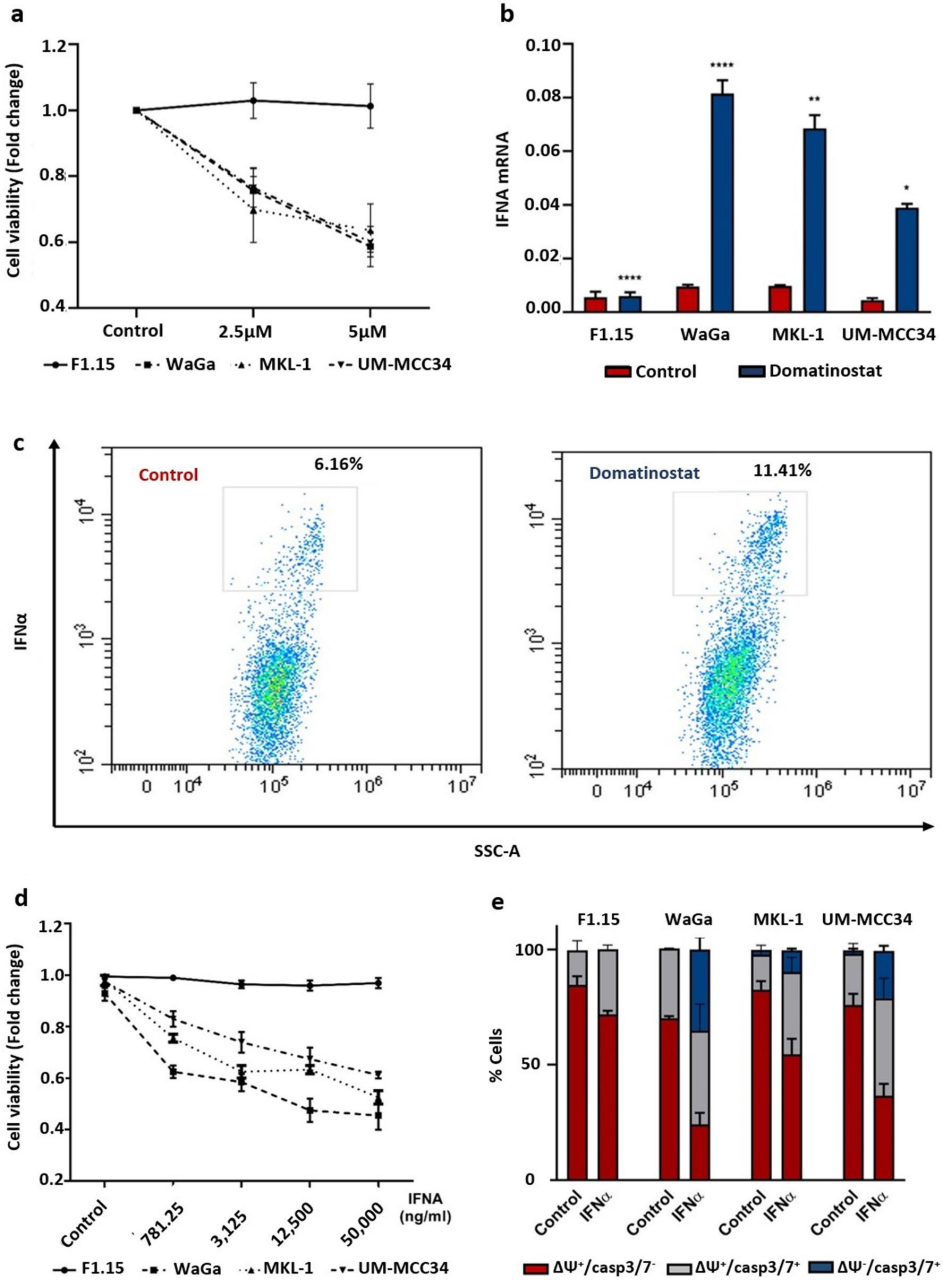
Induction of IFNα expression and apoptosis in MCC cells by domatinostat. **a** Viability of MCC cells (WaGa, MKL-1 and UM-MCC34) and primary fibroblasts treated with 2.5 µM and 5 µM domatinostat for 24 h at 37 °C was measured using CellTiter 96 AQueous One Solution Cell Viability Assay (Promega, Mannheim, Germany), absorbance was measured at 490 nm using a Spectramax microplate reader (Molecular Devices, San Jose, CA). **b** *IFNA* mRNA expression, in MCC cell lines and primary fibroblasts treated with 2.5 µM domatinostat for 24 h at 37 °C, was quantified by RT-qPCR using SYBR green assay. Relative quantification normalized to *RPLPO*, calculated by the 2^−ΔΔCt^ method, is depicted as mean + SD (*, *p* < 0.05; **, *p* < 0.01; ***, *p* < 0.001; ****, *p* < 0.0001). All the experiments were repeated at least twice. **c** Intracellular IFNα expression in WaGa cells after 6 h of treatment with domatinostat or solvent control in the presence of brefeldin A was quantified by flow cytometry. The values on the dot plots indicate the percentage of cells stained positive for IFNα. The experiment was repeated three times. **d** Viability of MCC cells and primary fibroblasts treated with the indicated concentrations of IFNα 2a for 7 days at 37 °C under was measured with CellTiter 96 AQueous One Solution Cell Viability Assay, absorbance was measured at 490 nm using a Spectramax microplate reader. **e** In the NucView 488/MitoView 633 Apoptosis Assay, healthy cells with an intact mitochondrial membrane potential are stained with MitoView633 (ΔΨm), while late apoptotic cells are stained with NucView488 (active caspase-3/7). MCC cells and primary fibroblasts were treated with 50,000 ng/ml IFNα 2a for 7 days at 37 °C. Visualized by flow cytometry and quantification is given as mean + SD and presented as stacked bars. All the experiments were repeated at least twice

Epigenetic mechanisms such as differential histone acetylation are involved in the regulation of IFN expression. Thus, HDACi is thought to upregulate production of type I IFNs, which in turn may be associated with apoptosis in malignant cells (Nusinzon and Horvath 2003; Lu et al. 2019; Kotredes and Gamero 2013). In this regard, it is important to note that we have observed in previous work that the class I selective HDACi domatinostat induces apoptosis of MCC cells (Song et al. 2021). To confirm this observation, we evaluated the effect of two doses of domatinostat (2.5 µM and 5 µM) on viability of MCC cells (WaGa, MKL-1 and UM-MCC34) and primary fibroblasts (F1.15). Within 24 h, domatinostat reduced the fraction of living MCC cells in a dose-dependent manner, while primary fibroblasts remained unaffected (Fig. 1a). To test whether domatinostat-induced cell death is associated with the induction of IFN, we measured the expression of *IFNA* mRNA. Indeed, we observed an increase in the expression of *IFNA* mRNA already in MCC cells treated with only 2.5 µM of domatinostat, while no induction was observed in primary fibroblasts (Fig. 1b). To confirm that the mRNA expression translated into protein expression, we performed intracellular IFNα staining in WaGa cells. We focused on this cell line because it grows in a single-cell suspension, as opposed to spheroids like the other cell lines, which makes these assays very difficult. Consistent with the recent report that IFN expression induced by MCPyV controls transcription of virally encoded early genes, we detected a constant number of IFNα-expressing cells in 3 independent experiments, which was approximately 5% (Wang et al. 2023). The frequency of these IFNα-expressing cells doubled in all experiments as early as 6 h after addition of domatinostat (Fig. 1c).

### IFNα inhibits MCC cell proliferation and induces apoptosis

In cancer cells in general and in MCC cells in particular, type I IFNs can both inhibit their proliferation and induce apoptosis (Willmes et al. 2012; Kotredes and Gamero 2013; Shi et al. 2022). In line with these reports, treatment of MCPyV-positive WaGa and MKL-1, as well as MCPyV-negative UM-MCC34 MCC cell lines with recombinant IFNα 2a resulted in a dose-dependent inhibition of cell proliferation (Fig. 1d). The cell viability was measured using CellTiter 96 AQueous One Solution Cell Viability Assay, as well as manually quantified based on Trypan blue exclusion. The effect of IFNα on the viability of MCC cells was at least in part due to the induction of apoptosis as detected by the loss of mitochondrial membrane potential (ΔΨm) and an increase in caspase-3/7 activity. A total of 35.1% of WaGa, 9.5% of MKL-1, and 20.4% of UM-MCC34 cells underwent apoptosis on treatment with 50,000 ng/ml IFNα for 7 days. We did not observe any effect on cell viability or induction of apoptosis in primary fibroblasts (Fig. 1e).

### Decreased expression of transcription factor HES1 by treatment with domatinostat

**Fig. 2:**
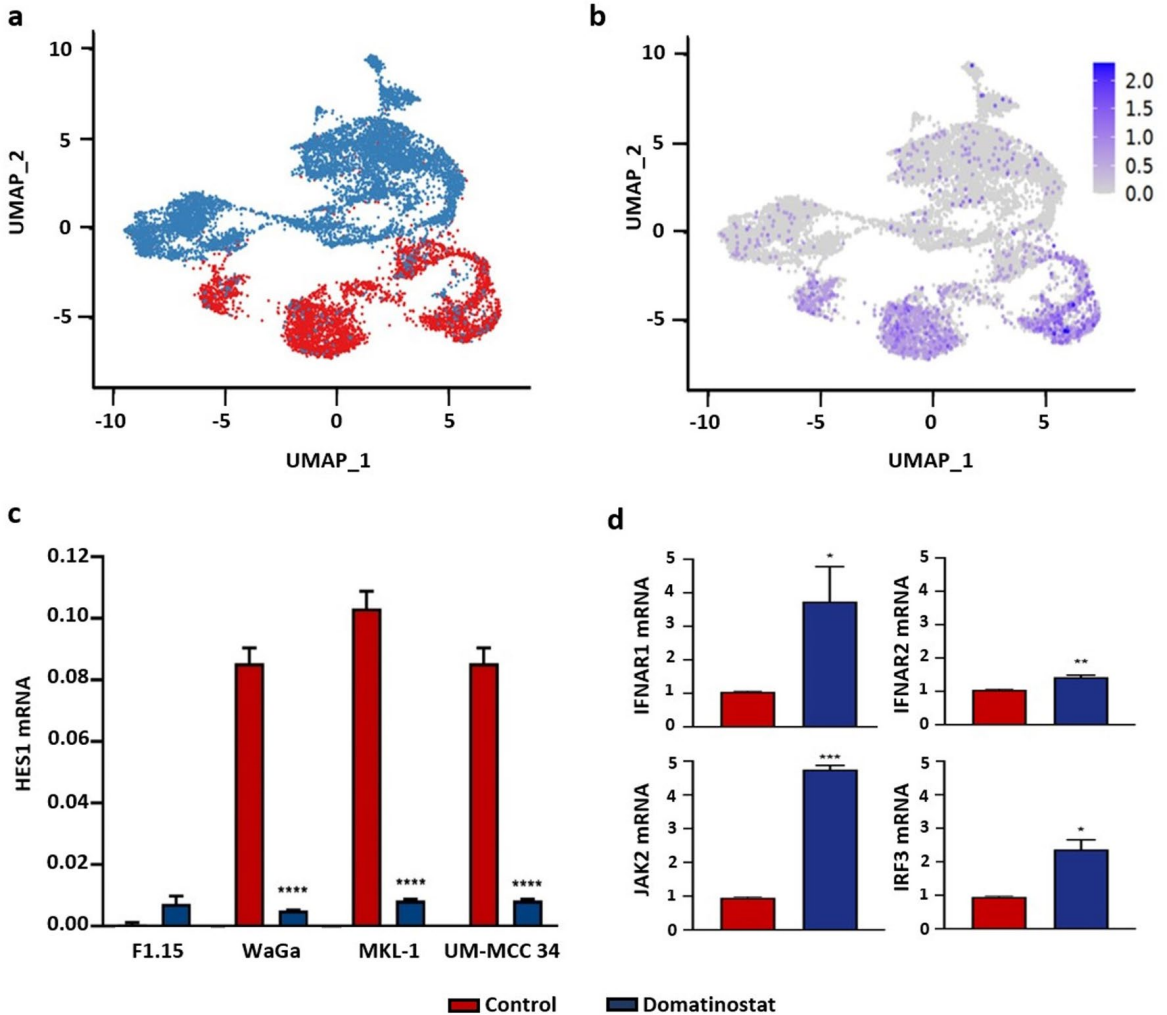
Domatinostat reduces the expression of transcription factor HES1. **a** Uniform Manifold Approximation and Projection (UMAP) of scRNAseq data from WaGa cells treated with domatinostat (blue) or not (red); normalized data were regressed for cell cycle effect. **b** In the same UMAP visualization as **a**, cells were annotated according to log-normalized HES1 expression. **c** *HES1* mRNA expression in the MCC cell lines- WaGa, MKL-1 and UM-MCC34, as well as, primary fibroblasts treated for 24 h at 37 °C with 2.5 µM domatinostat were quantified by RT-qPCR. **d** mRNA expression of interferon-stimulated genes *IFNAR1*, *IFNAR2*, *JAK2*, and *IRF3* in WaGa cells treated for 24 h at 37 °C with 2.5 µM domatinostat were quantified by RT-qPCR. Relative quantification normalized to *RPLPO* calculated by the 2^−ΔΔCt^ method is depicted as mean + SD (****, *p* < 0.0001). All the experiments were repeated at least twice

In our previous report, we scrutinized the effect of domatinostat treatment on the transcriptome of the MCC cell line WaGa by single-cell RNA sequencing (Song et al. 2021). After regressing for cell cycle effects, normalized data was visualized by Uniform Manifold Approximation and Projection (UMAP). This showed that the transcription factor HES1, a homeostatic negative regulator of type I IFNs, was downregulated upon domatinostat treatment (Ning et al. 2019) (Fig. 2a, b). This observation was confirmed in an independent series of experiments using additionally the MCPyV-positive MKL-1 and -MCPyV-negative UM-MCC34 cell lines quantifying HES1 mRNA expression by RT-qPCR (Fig. 2c). Because domatinostat increased IFN α mRNA and protein expression in MCC cells (Fig. 1b, c), we also determined mRNA expression of interferon-induced genes (ISG) in domatinostat-treated WaGa cells and observed an upregulation of *IFNAR1, IFNAR2, JAK2*, and *IRF3* (Fig. 2d).

### HES1 silencing induces IFNA and IFN-stimulated genes

**Fig. 3:**
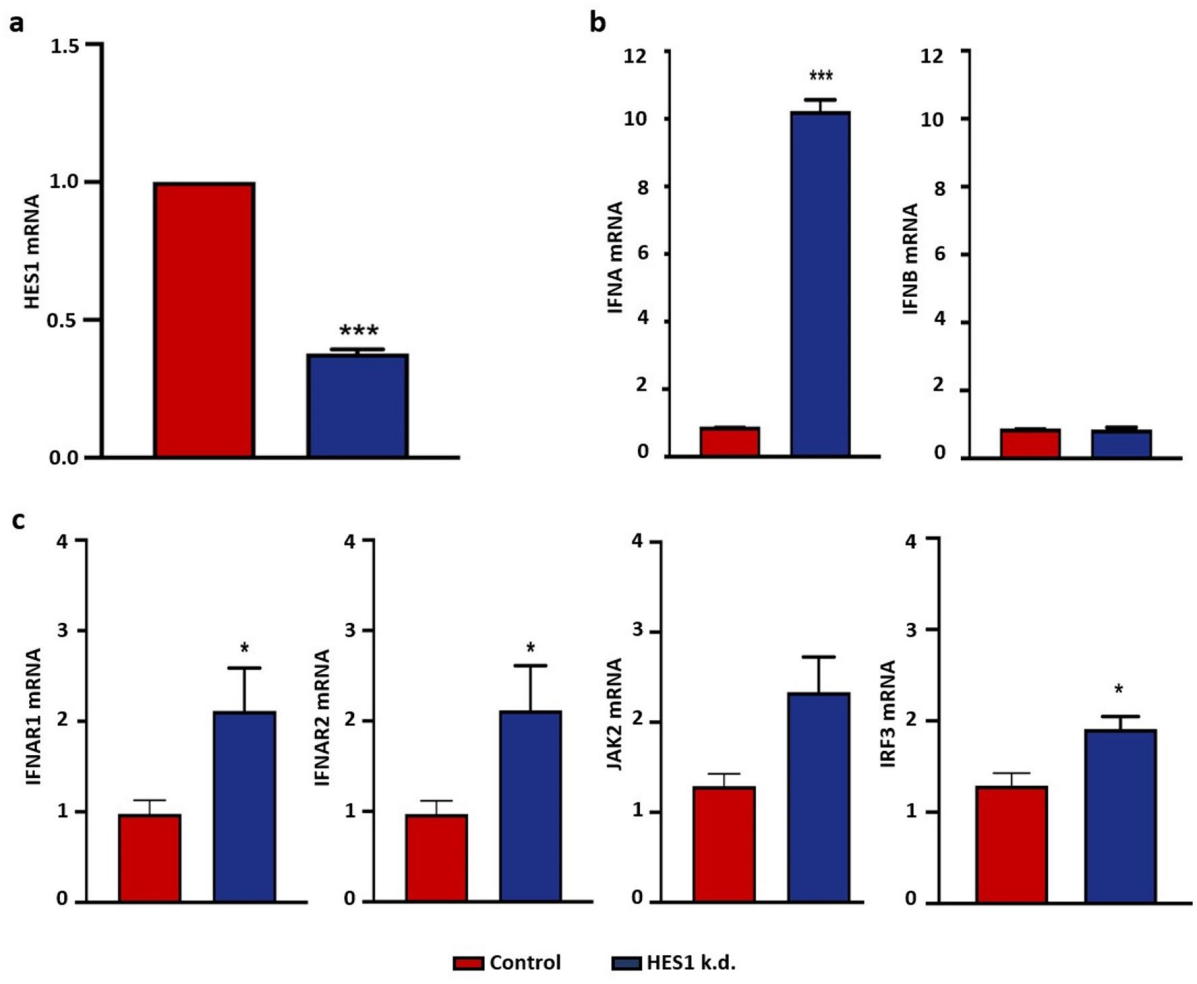
HES1 repression and induction of IFNα in MCC cells. **a** HES1 knockdown was performed in WaGa cells using dicer-substrate RNAi (TriFECTa kit, Integrated DNA Technologies, Leuven, Belgium) and Lipofectamine™ RNAiMAX (Thermofischer, Dreieich, Germany) with HES1 specific siRNA (HES1 k.d.) or scrambled control siRNA (control). *HES1* mRNA expression was measured 36 h post-transfection by RT-qPCR using SYBR. **b** mRNA expression of *IFNA* and *IFNB* and interferon-stimulated genes, *IFNAR1*, *IFNAR2*, *JAK2*, and *IRF3* was quantified in WaGa cells 36 h after HES1 knockdown or scrambled control by RT-qPCR. Relative quantification normalized to *RPLPO*, calculated by the 2^−ΔΔCt^ method, is depicted as mean + SD (*, *P* < 0.05; **, *P* < 0.01***, *p* < 0.001). All the experiments were repeated at least twice

To confirm that domatinostat-induced IFNα induction was indeed due to the observed suppression of HES1, the transcription factor was silenced using RNA interference. Predesigned dicer-substrate small interfering RNA against HES1 (hs.Ri.HES1.13.1) transfected into WaGa cells resulted in knockdown (k.d.) of HES1 mRNA by approximately 44%–62%, with maximum efficiency achieved at 36 h after transfection (Fig. 3a). The cell viability of transfected WaGa cells was reduced upon HES1 knockdown; however, we did not observe induction of apoptosis (data not shown). Albeit, HES1 is a negative transcriptional regulator of type I IFNs, i.e., IFNα and IFNβ, we only observed a significant increase in the expression of *IFNA*, but not *IFNB* upon HES1 k.d. (Fig. 3b). Importantly, the HES1 k.d.-mediated expression of IFNA mRNA is functional within the MCC cells as we observed an activation of downstream signaling pathways leading to expression of IFN-stimulated genes (ISGs), i.e., an upregulation of *IFNAR1*, *IFNAR2*, *JAK2*, and *IRF3* in HES1 knocked-down WaGa cells (Fig. 3c).

## Discussion

HDACs are often aberrantly overexpressed, predominantly leading to the transcriptional repression of tumor suppressor genes. Thus, HDACi are powerful drugs, with some already approved for certain hematological cancers such as cutaneous T cell lymphoma (CTCL) (Lopez et al. 2018). Induction of apoptotic cell death is one of the main mechanisms of the anticancer effect of HDACi; the underlying mechanisms depend on both the cellular context and HDACi itself. In this context, it should be noted that domatinostat not only selectively targets HDAC 1, 2 and 3, i.e., class I HDACs, but also inhibits lysine-specific histone demethylase 1A (LSD1) and RE1 silencing transcription factor (REST) already at low µM concentrations (Inui et al. 2017; von Tresckow et al. 2019). In particular, dysregulation of REST expression has been associated with neuroendocrine differentiation of MCC cells. Moreover, domatinostat strongly stimulates transcription of APM component genes in MCC cells that do not undergo apoptosis, resulting in enhanced surface expression of HLA class I (Song et al. 2021). Here, we add the induction of IFNα via domatinostat-mediated inhibition of HES1 as a mechanism that explains both effects.

We have previously demonstrated that domatinostat induces both G2M arrest and apoptotic cell death in MCC cell lines. Since the cell cycle inhibitor nocodazole induces complete G2M arrest in MCC cells but hardly any apoptosis, additional mechanisms for the proapoptotic activity of domatinostat than only G2M arrest were suspected (Song et al. 2021). Similarly, the rapid apoptotic effect of domatinostat, evident as early as 24 h after treatment, is also indicative for alternative mechanisms, given the long cell cycle times of 48 to 72 h for MCC cells (Schrama et al. 2019). In MCC cells treated with domatinostat that did not undergo apoptosis, we observed upregulation of molecules involved in antigen presentation, including the antigen processing-associated transporter (TAP) and proteasome subunits that generate peptides for MHC class I loading, which are normally regulated by type I IFNs. Because of the essential function of HDACs in modulating immune responses, the influence of HDACi on the regulation of type I IFNs has already been addressed in several studies, showing downregulation in some studies and upregulation in others (Salvi et al. 2010; Yang et al. 2022; Li et al. 2016). The latter may be explained by the fact that the expression of IFNs in cancer cells can be either constitutive or inducible, depending on the cell type and the specific molecular mechanisms involved (Cheon et al. 2023). We have previously shown the expression of IFNs in MCC cell lines on mRNA level and now confirmed it on protein level by intracellular cytokine staining (Paulson et al. 2011). It was recently reported, that MCPyV induces the expression of type I IFNs, which in turn stimulate robust expression ISGs (Wang et al. 2023). This induction does not appear to be critical for the control of viral replication, but rather represses the transcription of early viral-encoded genes. Such constitutive expression appears to particularly promote autocrine IFN signaling (Silginer et al. 2017). This constitutive IFN α expression may thus also explain for the high number of apoptotic MCC cells observed both in vitro and in situ (Mori et al. 2001). The number of IFNα-expressing cells is significantly increased—actually doubled—as early as 6 h after domatinostat treatment, explaining the early observed onset of apoptotic cell death after HDAC inhibition. The critical importance of induction of apoptosis by IFNα in the control of malignant transformed and virus-infected cells is well established (Thyrell et al. 2002; Peteranderl and Herold 2017).

Notably, the fraction of IFNα-expressing cells was not exceeding 10%. In this respect, it is important to note that previous studies, both in vitro and in vivo, have demonstrated that IFNs upon toll-like receptor-induced activation are induced in only a small fraction (i.e., 1–3% of the total population) of cells, also known as ‘early responding cells’. Subsequently, the IFNs produced prime a larger fraction of the surrounding cells via paracrine signaling to further enhance the IFN production, also known as ‘second responders’ (Van Eyndhoven et al. 2021). Since it has been previously shown that domatinostat can lead to phenocopy of acute viral infection in cancer cells, this early phase of IFN induction in still few cells is better captured by intracellular staining than by its secretion into the supernatant (von Tresckow et al. 2019; Silginer et al. 2017).

As viral infections are known to cause hyperacetylation of histones H3 and H4 at the promoter of IFNs, we assumed that the observed induction of IFNα by domatinostat is unlikely to be caused by direct chromatin remodeling. Revisiting of previous single-cell RNA sequencing data on WaGa cells, treated with domatinostat, revealed a decreased expression of the basic helix–loop–helix transcription factor HES1, a homeostatic negative regulator of type I IFNs (Shang et al. 2016). HES1 also functions as a transcription repressor by interacting with HDACs. Conversely, inhibition of HDAC activity by HDACis results in a more open chromatin conformation and transcriptional activation of target genes, which in turn, partially represses the effects of HES1 (Sang et al. 2010). We not only confirmed the reduced expression of *HES1* upon HDACi in an independent series of experiments, but also demonstrated that k.d. of *HES1* in MCC cells resulted in functional induction of IFNα by an activated IFN signaling. Since the apoptotic effect of external IFNα is much slower than after treatment with domatinostat, the latter appears to alter the state of MCC cells in yet another way. As a primary target of Notch signaling pathway, HES1 has previously been shown to regulate tumor cell proliferation and survival, and silencing HES1 causes both growth arrest and apoptosis (Kobayashi and Kageyama 2014; Huang et al. 2018). In line with this, HES1 k.d. impaired the viability of WaGa cells, however, we did not observe an induction of apoptosis.

HES1 functions as a homeostatic negative regulator of type I IFNs essential for the maintenance of the immune balance (Ning et al. 2019; Shang et al. 2016). It inhibits toll-like receptor (TLR)-mediated induction of type I IFNs and thus downstream signaling pathways otherwise leading to the transcriptional induction of ISGs (Marie et al. 2018; Ivashkiv and Donlin 2014). Notably, HES1 does not directly suppress type I IFNs, but rather through regulating the expression of TLR signaling adaptor protein, WDYF1, via modulating its upstream inhibitory factor, VEGF-C (Ning et al. 2019; Hu et al. 2015). Some studies have also reported a reciprocal regulation between HES1 and TLR signaling pathways, where HES1 participates in and regulates TLR responses, such as IFN production (Hu et al. 2008; Zhang et al. 2012). This heightens the importance of investigating the HES1-IFN axis, both functionally, as well as mechanistically in our present study. TLRs are integral membrane proteins of the innate immune system and activate pathways that not only lead to IFNα production, but also trigger downstream signaling pathways influencing which ISGs are activated or repressed (Kawai and Akira 2010; McNab et al. 2015). IFNα binds to a heterodimeric transmembrane receptor—the IFNα receptor—that activates JAK/STAT signaling pathway and further downstream effectors such as interferon response factors (IRFs) (Zanin et al. 2020). We observed an upregulation of the ISGs- *IFNAR1, IFNAR2, JAK2,* and *IRF3* both upon domatinostat treatment and after *HES1* k.d. Moreover, HES1 physically interacts with downstream genes of type I IFN signaling by facilitating complex formation between JAK2 and STAT3, promoting STAT3 phosphorylation and activation (Cenciarelli et al. 2017). Normal cells express infrequent and low levels of ISGs, whereas interferon produced by cells in the tumor microenvironment, as well as by the tumor cells themselves, induces their expression (Cheon et al. 2023). The induced ISG profile may ultimately dictate the cellular response to IFNα signaling mediated by HES1 suppression in WaGa cells. Concurrently, the ability of IFNα to inhibit cellular proliferation or induce apoptosis is largely attributable to the induction of ISGs (Peteranderl and Herold 2017; McNab et al. 2015).

In summary, our results indicate that the direct anti-tumor effect of HDACi domatinostat is mediated, at least in part, by downregulation of HES1, leading to induction of IFNα, which in turn induces apoptosis in MCC cells. However, whether downregulation of HES1 is the only mechanism mediating the effect of HDACi requires further validation.

## Data Availability

Datasets of single-cell RNA sequencing of the Merkel cell carcinoma cell line WaGa on domatinostat treatment compared with those of untreated WaGa control cells were uploaded to the National Center for Biotechnology Information Gene Expression Omnibus (GSE10346).
